# Hyperbaric oxygen therapy for knee osteoarthritis: treatment mechanism, potential advantages, and limitations

**DOI:** 10.4103/mgr.MEDGASRES-D-25-00147

**Published:** 2026-04-11

**Authors:** Xueting Zhou, Hairui Liang, Hanfei Liu, Ming Sun, Zhencun Cai

**Affiliations:** 1 Department of Orthopedics Surgery, The Second Affiliated Hospital of Shenyang Medical College, Shenyang, Liaoning Province, China; 2 Department of Orthopedic Surgery, Central Hospital Affiliated to Shenyang Medical College, Shenyang, Liaoning Province, China; 3 Liaoning Province Key Laboratory for Phenomics of Human Ethnic Specificity and Critical Illness, and Shenyang Key Laboratory for Phenomics, Shenyang, Liaoning Province, China

**Keywords:** angiogenesis, cartilage repair, chondrocyte, combination therapy, hyperbaric oxygen therapy, hypoxia, inflammation, knee osteoarthritis, oxidative stress, precision medicine

## Abstract

Knee osteoarthritis is a common chronic joint disease. Although there are many treatment options available for knee osteoarthritis, most focus on alleviating symptoms rather than halting disease progression, repairing cartilage, reversing joint damage, or restoring the structure and function of the knee. Hyperbaric oxygen therapy works by increasing tissue oxygen concentrations by increasing higher atmospheric pressure. This therapy can directly improve the hypoxic microenvironment in knee osteoarthritis. Hyperbaric oxygen therapy can also improve oxygenation, reduce oxidative stress and regulate inflammatory responses. In addition, it plays an important role in promoting angiogenesis, cartilage repair and regeneration, and healing of damaged joint tissue. This review explores the therapeutic effect of hyperbaric oxygen therapy on knee osteoarthritis, systematically analyzes its therapeutic mechanism, potential advantages and current limitations, and provides direction for future research. As it continues to develop, hyperbaric oxygen therapy has the potential to become a non-invasive, efficient and highly targeted treatment option, bringing new hope to patients with knee osteoarthritis.

## Introduction

As the most common chronic degenerative joint disease in the world, the incidence of knee osteoarthritis (KOA) increases with age, with a higher prevalence in women than in men.^1^ More than half of new KOA cases occur before the age of 55, and early-onset disease accounts for about one-quarter of total disability burden, with the incidence nearly doubling since 1990, especially in countries with low and middle sociodemographic indexes. Women show a consistently higher prevalence than men, with sex-specific risk factors such as high body mass index and alcohol use in women and abdominal obesity and high-intensity activity in men. Symptom severity is influenced by sleep, comorbidities, and psychological factors rather than structural damage alone, and longitudinal data indicate that while most patients remain stable over 10 years, women and those with multimorbidity are more likely to experience progressive disability.^2^,^3^

The pathological progression of KOA is closely related to the hypoxic microenvironment. Chondrocytes, which are the functional units of avascular tissues, heavily rely on diffusion for oxygen supply in their metabolism. In the early stages of KOA, synovitis and osteophyte formation occur, leading to a decrease in intra-articular oxygen partial pressure, which triggers mitochondrial dysfunction and apoptosis in chondrocytes.^4^,^5^ Traditional drugs, such as non-steroidal anti-inflammatory drugs can only suppress inflammation but cannot reverse hypoxia-induced cell death.

Hyperbaric oxygen therapy (HBOT), as a non-invasive physical therapy, has shown a gradual expansion in its application within the field of orthopedics in recent years. The core mechanism of HBOT involves alleviating the hypoxic microenvironment of tissues, inhibiting inflammatory responses, and promoting chondrocyte repair.^6^ However, research on HBOT remains insufficient, and the specific mechanisms for treating KOA, as well as the applicable patient population and standardized treatment protocols, still require further investigation. Therefore, this review aims to systematically summarize the current evidence on HBOT in the treatment of KOA, elucidate its underlying mechanisms, identify the potential patient population, and highlight the gaps in standardized treatment protocols to guide future research and clinical practice.

## Literature Retrieval

A systematic literature search was conducted to comprehensively summarize the progress in both basic and clinical research on HBOT for the treatment of KOA. The entire process strictly followed the Preferred Reporting Items for Systematic Reviews and Meta-Analyses (PRISMA) guidelines^7^ to ensure methodological rigor, transparency, and reproducibility.

The literature search was conducted across multiple databases, including PubMed/MEDLINE, Web of Science, Scopus, CNKI (to supplement Chinese-language publications), and the Cochrane Library (for systematic reviews and meta-analyses). The search covered publications from January 1, 2000 to June 30, 2025, with the last search completed on June 30, 2025. Both English and Chinese literature were included to capture a comprehensive overview of global research progress.

The search strategy integrated both free-text keywords with Medical Subject Headings (MeSH) terms, constructing search queries using Boolean operators (AND/OR): (“Knee Osteoarthritis”[MeSH Terms] OR “KOA”) AND (“Hyperbaric Oxygenation”[MeSH Terms] OR “HBOT” OR “Hyperbaric Oxygen Therapy”) AND (“Treatment Outcome”[MeSH Terms] OR “Angiogenesis” OR “Chondrocyte metabolism”). Additional terms such as “hypoxia” and “cartilage metabolic dysfunction” were also considered based on terminology found in key publications.

Studies were included if they met the following criteria: (1) involving KOA patients or animal models; (2) describing HBOT explicitly as a treatment modality, either alone or in combination with other therapies; (3) reporting outcomes such as therapeutic efficacy, mechanisms of action, or adverse effects; (4) providing full-text access in English or Chinese; and (5) original research articles, systematic reviews, or meta-analyses. Exclusion criteria were as follows: (1) incomplete data or lack of clearly reported outcomes; (2) non–peer-reviewed publications; and (3) duplicate studies.

**Figure 1:**
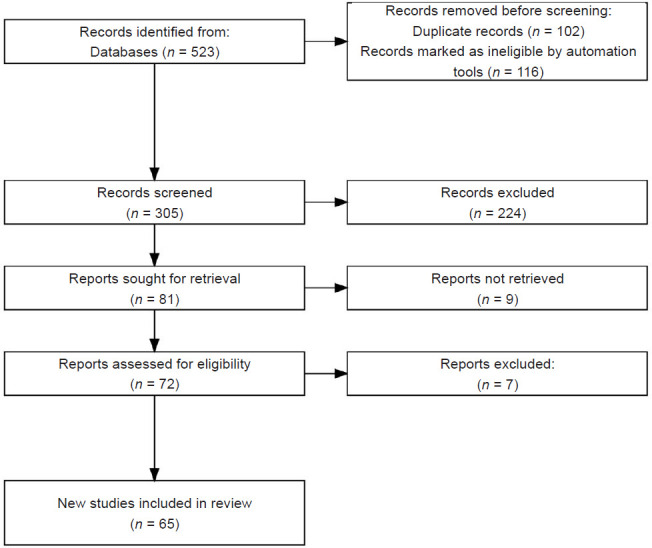
Literature search flowchart. Created with shinyapps.io.^8^

Based on this comprehensive search strategy, relevant high-quality literature published between 2000 and 2025 was systematically retrieved and screened. Older publications were also included when necessary to provide historical context or supplement mechanistic insights. The entire screening process is illustrated in the PRISMA flow diagram (**Figure 1**).^8^

## Knee Osteoarthritis

### Pathophysiological mechanisms of knee osteoarthritis

The main features of KOA are the wear of articular cartilage, remodeling of subchondral bone, osteophyte formation, and peripheral inflammation. Some patients also experience ligament disruptions and partial muscle dysfunction. From a pathological perspective, the hallmark of KOA is the imbalance between the synthesis and degradation of the extracellular matrix in chondrocytes. This imbalance process is not only associated with chronic inflammation but also influenced by hypoxia.^9^,^10^,^11^

#### Articular cartilage destruction and inflammatory factor stimulation

The earliest pathological changes of KOA occur at the surface of the articular cartilage, which is immersed in the extracellular matrix.^11^,^12^ The components of the extracellular matrix include water, proteoglycans, minerals, and a collagen fiber network.^11^ Under mechanical stimulation, chondrocytes, proteoglycans, and collagen in the cartilage of KOA patients proliferate. Similarly, the synthesis of inflammatory cytokines, such as interleukin (IL)-1 and tumor necrosis factor-α (TNF-α), is increased. These cytokines stimulate the production of matrix metalloproteinases, aggrecans (especially a disintegrin and metalloproteinase with thrombospondin motifs-4 and 5), nitric oxide (NO), prostaglandins, and leukotrienes, all of which contribute to the destruction of joint tissues.^12^,^13^,^14^,^15^

#### Hypoxia

Due to the lack of blood vessel distribution, cartilage tissue is often in a state of hypoxia. In this hypoxic environment, the level of reactive oxygen species (ROS) in chondrocytes increases, leading to enhanced activity of matrix metalloproteinases, which accelerates the degradation of extracellular matrix.^16^,^17^

Hypoxia-inducible factors (HIFs) play an important role in maintaining chondrocyte function and cartilage tissue homeostasis. Among them, HIF-1α is particularly critical. It can not only regulate the metabolic activities of chondrocytes, but also promote early cartilage formation. Upregulation of HIF-1α expression affects the balance between extracellular matrix synthesis and degradation through two major ways: on the one hand, HIF-1α promotes the transcription of pro-angiogenic factors such as vascular endothelial growth factor (VEGF), thereby promoting blood vessel invasion into cartilage tissue and affecting cartilage ossification and remodeling process; on the other hand, it can also affect the stability of the matrix by changing the expression level of matrix-degrading enzymes.^18^

Cartilaginous damage often leads to tissue edema and hypoxia, which in turn leads to an increase in levels of pro-inflammatory factors such as IL-1 and TNF-α. These cytokines not only stimulate the expression of matrix metalloproteinases and intensify the degradation of extracellular matrix, but also directly or indirectly promote the transcriptional activity of HIF-1α, forming a vicious cycle and breaking the metabolic balance of chondrocytes. Multiple mechanisms work together to ultimately promote the occurrence and development of knee osteoarthritis.^19^,^20^

The hypoxic environment also affects the metabolism of chondrocytes. The energy source of chondrocytes is the glycolytic pathway, and under hypoxic conditions, the rate of glycolysis, intracellular adenosine triphosphate levels, and extracellular lactate secretion significantly decrease. With energy metabolism affected, the repair and regeneration capacity of cartilage may also be impaired.^17^,^21^

**Figure 2:**
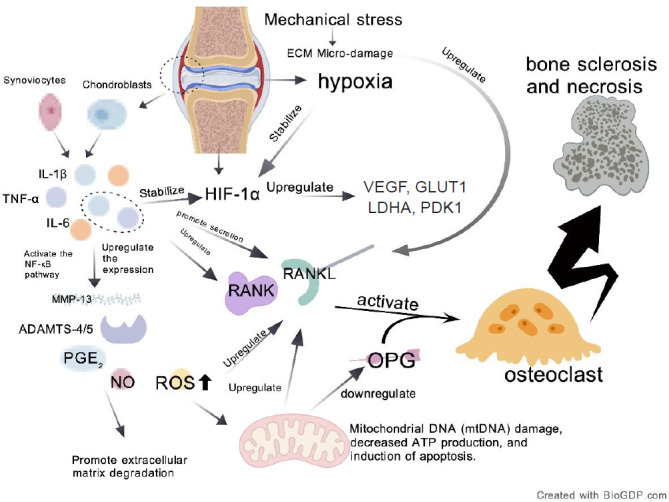
Pathophysiology of KOA. Created with BioGDP.com.^22^ ADAMTS-4/5: A disintegrin and metalloproteinase with thrombospondin motifs-4 and 5; ECM: extracellular matrix; GLUT1: glucose transporter 1; HIF-1α: hypoxia-inducible factor 1α; IL: interleukin; KOA: knee osteoarthritis; LDHA: lactate dehydrogenase A; MMP-13: matrix metalloproteinase-13; mtDNA: mitochondrial DNA; NF-κB: nuclear factor κB; NO: nitric oxide; OPG: osteoprotegerin; PDK1: pyruvate dehydrogenase kinase 1; PGE2: prostaglandin E2; RANK: receptor activator of NF-κB; RANKL: RANK ligand; ROS: reactive oxygen species; TNF-α: tumor necrosis factor-α; VEGF: vascular endothelial growth factor.

The occurrence and development of knee osteoarthritis involve multiple mechanisms, which are intertwined and inseparable (**Figure 2**).^22^

### Current treatments and limitations for knee osteoarthritis

#### Pharmacological treatment

Weight management is an important component of KOA treatment. Weight loss has been shown to alleviate symptoms of KOA. Bliddal et al.^23^ found that obese patients who used Semaglutide in combination with dietary control significantly reduced their body weight (–13.7%) and pain (Western Ontario and McMaster Universities Osteoarthritis Index decreased by 41.7 points), although the probability of gastrointestinal side effects was not negligible. In terms of pain relief, methotrexate may help alleviate pain, stiffness, and dysfunction in KOA patients, but the hepatotoxic risk associated with oral methotrexate must also be considered.^24^ To address the potential rapid joint damage and cystic lesions at the osteochondral junction in KOA patients, Au et al.^25^ confirmed through animal experiments using the golden Syrian hamster model that the endothelin receptor antagonist Macitentan inhibits cartilage degeneration. They also histologically confirmed the activation of osteoclasts, loss of chondrocytes, and cyst formation.

#### Rehabilitation treatment

In terms of exercise interventions, Jacobs et al.^26^ demonstrated in a randomized controlled trial that incorporating blood flow restriction training into traditional exercise therapy significantly reduced knee pain in the subjects, alleviated symptoms, and improved function. The addition of blood flow restriction allowed metabolic stimulation necessary for muscle strength and function gains at lower mechanical loads.

Following the European Alliance of Associations for Rheumatology guidelines, Moseng et al.^27^ recommended considering the addition of customized insoles and walking aids based on appropriate exercise and weight loss.

#### Biological treatment

To date, various types of cell injections have become a popular and costly treatment option for KOA patients. Mautner et al.^28^ conducted a multicenter, randomized controlled trial comparing the safety and efficacy of autologous bone marrow aspirate concentrate, autologous adipose-derived stromal vascular fraction, and allogeneic human umbilical cord-derived mesenchymal stromal cell injections with corticosteroid injections in the treatment of KOA. After 1 year of injections, none of the three cell injection methods showed superior efficacy to each other or corticosteroid injection. This suggests that although cell injections theoretically have the potential to repair cartilage, their efficacy in practical applications is not superior to traditional corticosteroid injections.

Huang et al.^29^ showed that magnetized cartilage extracellular matrix-derived scaffolds, a decellularized natural pig cartilage extracellular matrix derivative, demonstrated better knee function recovery in clinical trials compared with the control group, as they have a biomechanical environment similar to human cartilage, can efficiently load functional cells, and spontaneously release cells at target sites.

Wang et al.^30^ explored gene and RNA therapy approaches and experimentally demonstrated that intra-articular injection of recombinant IL-6 receptor or activation of gp130 (Y757F mutation) accelerates the progression of KOA, whereas knockout of IL-6 receptor or Janus kinase 2 or use of Janus kinase inhibitors alleviates symptoms. They also conducted mechanistic studies and confirmed that Janus kinase inhibition can stabilize Sirtuin 1 reduce acetylation of sex-determining region Y-box 9 (SOX9), promote SOX9 nuclear localization, and thereby promote cartilage repair. In addition, Janus kinase inhibitor-induced p21-positive senescent cell apoptosis and targeted clearance can effectively alleviate the progression of KOA.

Zhao et al.^31^ found that fibroblast activation protein expression was significantly increased in the cartilage of KOA patients and was associated with chondrocyte senescence and disease progression. Silencing fibroblast activation protein using small interfering RNA technology can reduce chondrocyte senescence, inhibit the nuclear factor κB (NF-κB) pathway, and decrease the senescence-associated secretory phenotype.

#### Surgical treatment

In 2023, the American College of Rheumatology and the American Hip and Knee Society released clinical practice guidelines on the optimal timing for hip and knee arthroplasty. The guidelines recommend that, for symptomatic and radiologically confirmed moderate to severe OA or end-stage symptomatic avascular necrosis with secondary hip or knee arthritis, total joint arthroplasty should be considered if non-surgical treatments are ineffective.^32^

In conclusion, there are currently multiple treatment options available for KOA, but the limitations of these treatments cannot be ignored. Medication can alleviate pain, but long-term use may lead to gastrointestinal side effects, increase cardiovascular risks, and raise concerns about analgesic addiction. The effectiveness of physical therapy varies from person to person, and it requires long-term adherence to maintain results, which imposes demands on patient compliance and finances, and it is difficult to maintain high treatment enthusiasm. Cell-based therapies also exhibit significant individual variability. Surgical treatment may seem like one-time solution, but factors such as surgical risks, anesthesia impact, postoperative recovery time, and high costs are important considerations for elderly patients, making surgery unsuitable as a universal therapy. Although biological treatments hold potential for disease modification, their safety and efficacy still require further validation.

#### Emerging technologies

Gao et al.^33^ combined artificial cell compartmentalization with the intelligent movement advantages of micro/nanomotors, using light-driven artificial cell micro-motors to promote adenosine triphosphate synthesis and improve cartilage metabolism. These emerging technologies such as nanomaterials and micro-robotics have shown promising results in experiments, but they are not yet widely available for clinical use due to complexity, high costs, and potential biological safety concerns, and large-scale trial validation is still lacking. The deficiencies of the above treatment methods all suggest that new therapeutic strategies are urgently needed.

## Hyperbaric Oxygen Therapy

### Mechanism of hyperbaric oxygen therapy

HBOT works through multiple mechanisms. It effectively inhibits the production of ROS, and relieves oxidative stress, thereby maintaining cell homeostasis.^34^ Hadanny et al.^35^ found that 40 consecutive HBOT treatments, each inhalation of pure oxygen at 2.0 atmospheres absolute (2.0 ATA) for 1 hour, significantly improved mitochondrial respiratory function and quality, helping maintain the integrity and functional stability of the mitochondrial network structure.

HBOT helps stabilize mitochondrial DNA. It brings mitochondrial gene transcription back to normal. The balance between mitochondrial fission and fusion improves. These effects work together to protect mitochondria in heart tissue under ischemic conditions. Cells become less prone to energy failure and death. Damage to mitochondrial DNA does not spiral out of control. The therapy interrupts the cycle of mitochondrial injury and helps restore function.^36^ HBOT can not only reduce inflammatory reactions, but also promote tissue repair and functional recovery through a combination of anti-inflammatory and anti-oxidation.^37^ HBOT raises tissue oxygen pressure. The extra oxygen relieves hypoxia in the tumor microenvironment. The change disrupts the Warburg effect, a glycolysis-driven metabolic shift fueled by low oxygen. Under hypoxic conditions, HIF-1α stays active. It turns on glycolytic genes like glucose transporter 1, lactate dehydrogenase A, and pyruvate dehydrogenase kinase 1. With higher oxygen levels, HIF-1α becomes unstable. Gene expression tied to glycolysis drops. Tumor cells rely less on glycolysis and begin to shift their metabolic patterns. In multiple animal models, the therapy boosts oxidative phosphorylation. Tumor cells show more signs of apoptosis. Blood vessel formation slows down. These shifts break the feedback loop of hypoxia and metabolic reprogramming.^38^ Tumor cells start to behave more like normal cells—less aggressive, more differentiated. HBOT shows promise as a support strategy for treating tumors that depend heavily on glycolysis.^38^

### Application of hyperbaric oxygen therapy

HBOT is a treatment method that intermittently inhales 100% pure oxygen in an environment of more than 1 atmosphere. It has been used in the medical field for more than a century and has demonstrated potential therapeutic capabilities in the treatment of a variety of diseases.

Rinaldi et al.^39^ focused on the effect of HBOT on Toll-like receptor signaling pathways in multiple organ dysfunction syndromes. HBOT at 2.5 ATA was administered once daily for 60 minutes. Thy found that HBOT significantly reduced yeast glucan-induced activation of the Toll-like receptor signaling pathway in multiple organ dysfunction syndromes. Specifically, HBOT could significantly reduce damage to the lungs, liver and small intestine. It could also significantly reduce the expression of Toll-like receptor 2 and Toll-like receptor 4, the activation of NF-κB, and the production of cytokines. These results suggest that HBOT may protect against tissue damage caused by systemic inflammation by interfering with Toll-like receptor signaling pathways.

Chen et al.^36^ focused on cerebral ischemia-reperfusion injury, and the results showed that HBOT (2.0 ATA, 60 minutes/day for 7 consecutive days) reduced the cognitive impairment induced by repeated cerebral ischemia-reperfusion injury by inhibiting autophagy. HBOT significantly improved learning and memory, reduced levels of autophagy markers such as microtubule-associated protein 1 light chain 3, type II and autophagy-related protein 5 in the prefrontal cortex, and activated mTOR and eukaryotic translation initiation factor 4E-binding protein 1 signaling pathways. Therefore, HBOT exerts neuroprotective effects by suppressing inflammatory responses, regulating calcium ion concentration and p53 levels, while simultaneously increasing the expression of brain-derived neurotrophic factor and phosphorylated protein kinase B. Gao et al.^40^ confirmed that pretreatment with HBOT can reduce oxidative stress and inflammatory reactions, and effectively prevent or improve postoperative cognitive dysfunction. Pretreatment with HBOT protects important organs such as the brain by activating endogenous antioxidant and anti-inflammatory defense systems.

HBOT promotes apoptosis of peripheral blood lymphocytes, thereby helping to relieve early symptoms of acute pancreatitis in rats. In an early-stage acute pancreatitis rat model, HBOT increased oxygen levels, manifested by increases in arterial oxygen saturation and arterial oxygen partial pressure.^41^ Blood tests showed decreased serum amylase and lipase, indicating reduced pancreatic damage. At the molecular level, HBOT regulates the expression of cytokines, including IL-2, interferon-γ and IL-10, thereby effectively suppressing the inflammatory response.

Chen et al.^42^ looked at a heart ischemia-reperfusion model. After the injury, the mitochondria did not work well. The cells lost mitochondrial DNA copies. ND1 gene activity dropped. This change meant the mitochondria could not handle oxidative phosphorylation properly. Pretreatment with HBOT (2.5 ATA, 60 minutes daily for 5 days) brought things back on track. The therapy restored mitochondrial DNA levels and ND1 expression. Oxidative phosphorylation picked up again. The results showed that the mitochondria stayed in better shape after the therapy.

HBOT has also shown potential in the treatment of other diseases. Akiya et al.^43^ found that the combination of HBOT (100% O_2_, 2.0 ATA, 60 minutes/session) and nimustine hydrochloride significantly inhibited the progression of bladder tumors induced by N-butyl-N-(4-hydroxybutyl)nitrosamine and reduced bladder weight, suggesting that HBOT may enhance the efficacy of chemotherapy drugs by improving the oxygenation of tumor tissue. Rose et al.^44^ pointed out that HBOT significantly reduces the neurological sequelae in carbon monoxide poisoning patients, although some survivors still experience significant morbidity. It indicated that HBOT alone cannot address the complex pathophysiological processes following carbon monoxide poisoning, and the addition of other treatments is essential. Clinical research by Dhamodharan et al.^45^ showed that HBOT (100% O_2_, 2.5 ATA, 90 minutes per session, 5 sessions per week for 4 weeks) can promote the expression of nuclear factor erythroid 2–related factor 2 and its downstream target genes, thereby promoting the healing of diabetic foot ulcers. Meanwhile, HBOT could also significantly increase the levels of angiogenesis markers (such as epidermal growth factor, vascular endothelial growth factor, platelet-derived growth factor, fibroblast growth factor-2, and C-X-C motif chemokine ligand 10) in ulcer tissues, while increasing the concentrations of endothelial nitric oxide synthase and nitrite. In addition, HBOT can activate macrophages, release fibroblast growth factor-2 and epidermal growth factor, thereby promoting angiogenesis, and can increase the level of C-X-C motif chemokine ligand 8 to promote new blood vessel formation. These mechanisms work synergistically to reduce inflammation and promote tissue repair.

## Clinical Application and Research Progress of Hyperbaric Oxygen Therapy in Knee Osteoarthritis

When chondrocytes are chronically exposed to hypoxic conditions, extremely low oxygen pressures (PO_2_ lower than 1%) can inhibit their metabolic functions. Although cartilage tissue has few blood vessels, its normal function relies on adequate blood supply. During cartilage repair, angiogenesis is crucial. HBOT increases oxygen solubility and tissue oxygen content, provides metabolic support for hypoxic cells, and promotes the formation of new blood vessels, thereby assisting in cartilage healing.^6^,^46^ Meanwhile, the accumulation of ROS damages the extracellular matrix of cartilage cells. By adjusting the local PO_2_, HBOT shows potential therapeutic advantages in promoting type II collagen generation and preventing excessive ROS accumulation, thus supporting cartilage repair. Nugud et al.^47^ showed that by regulating ROS, HBOT not only prevents the death of osteoblasts and chondrocytes, but also optimizes signal transduction pathways inside and outside cells. These factors together promote angiogenesis, improve the microenvironment of severe hypoxia in the cartilage, promote the growth and differentiation of fibroblasts, and ensure efficient synthesis of extracellular matrix components such as collagen.^48^

HBOT protects mitochondria, reduces ROS buildup, lowers risks of inhibited bone formation and chondrocyte death, and preserves normal chondrocyte metabolism. By accurately regulating local PO_2_, HBOT controls inflammation effectively. It boosts fibroblast proliferation and differentiation by supplying enough oxygen, enhancing collagen synthesis and creating an optimal environment for cartilage repair.^34^,^42^ Existing studies have demonstrated that HBOT can enhance PHD activity by increasing oxygen partial pressure, thereby reducing HIF-1α expression and downregulating its downstream glycolytic target genes.^38^

HBOT has significant anti-inflammatory effects. A systematic review by de Wolde et al.^34^ emphasized that HBOT regulates the NF-κB/inhibitor of kappa B alpha (IκBα) axis by increasing the partial pressure of intracellular oxygen. This stably inhibits IκBα, prevents its degradation and inhibits nuclear translocation of NF-κB p65/p50, thereby inhibiting the expression of pro-inflammatory cytokines such as TNF-α and IL-1β. This mechanism may explain the clinically observed decrease in systemic inflammation biomarkers such as C-reactive protein and TNF-α. In line with this, Sun et al.^49^ reported that in an animal model of acute spinal cord injury, HBOT significantly reduced the nuclear translocation of NF-κB and the activation of the high mobility group protein B1 (HMGB1)/NF-κB pathway by up-regulating the IκBα, thereby reducing inflammation and tissue damage. Although HBOT has not been clearly demonstrated to directly inhibit NF-κB activation, studies on the mechanism of bone tissue repair suggest that it may indirectly regulate NF-κB activity through the receptor activator of nuclear factor kappa-beta ligand (RANKL)/RANK/osteoprotegerin (OPG) axis. It was found that HBOT could significantly increase serum OPG levels, while RANKL expression changed little, thereby increasing the OPG/RANKL ratio.^50^ Given that OPG, as a decoy receptor, can competitively inhibit the binding of RANKL and RANK, it is speculated that HBOT may use this mechanism to reduce the activation frequency of the NF-κB pathway, thereby inhibiting osteoclast activity, and ultimately promoting bone regeneration.^51^,^52^

HBOT interacts with extracellular vesicles and non-coding RNA while inhibiting inflammatory pathways. After hypoxia pretreatment, the extracellular vesicles secreted by mesenchymal stem cells have a better anti-inflammatory effect, which not only reduces IL-1α induced damage, but also protects cartilage.^53^ HBOT regulates RNA expression in chondrocytes, including reducing microRNA-107 levels, thereby inhibiting the HMGB1/receptor for advanced glycation end-products signaling pathway, ultimately reducing inflammatory responses and cartilage degradation.^54^

HBOT also activates protective signaling pathways such as phosphoinositide 3-kinase/protein kinase B, which resists IL-1β-induced apoptosis, maintains chondrocyte survival and ensures the integrity of the extracellular matrix. These synergistic effects promote cartilage protection and have been confirmed in *in vivo* and *in vitro* studies.^55^,^56^,^57^

**Figure 3:**
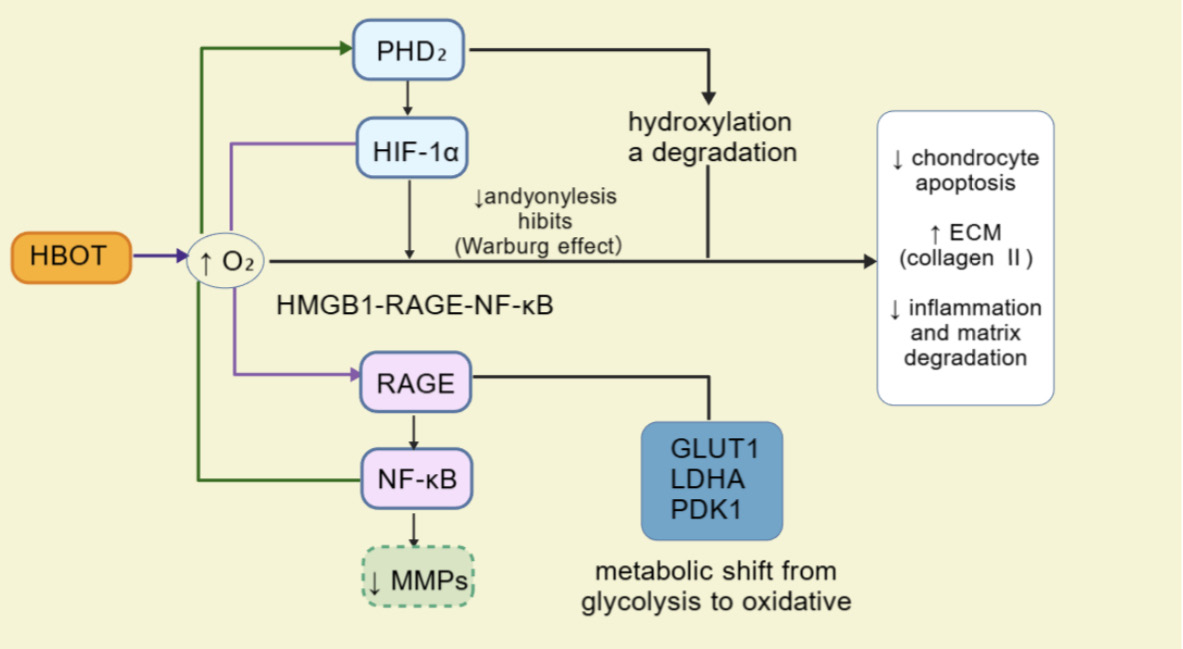
Mechanism of HBOT regulating the hypoxia–inflammation–metabolism network in the cartilage. Created with BioGDP.com.^22^ ECM: Extracellular matrix; GLUT1: glucose transporter 1; HBOT: hyperbaric oxygen therapy; HIF-1α: hypoxia-inducible factor 1α; HMGB1: high mobility group protein B1; LDHA: lactate dehydrogenase A; MMP: matrix metalloproteinase; NF-κB: nuclear factor κB; O_2_: oxygen; PDK1: pyruvate dehydrogenase kinase 1; PHD_2_: hypoxia-inducible factor prolyl hydroxylase 2; RAGE: receptor for advanced glycation end-products.

The cartilage repair mechanism of HBOT involves multiple regulatory effects that not only help reduce the release of inflammatory factors, but also optimize the microenvironment of cartilage repair and provide multi-faceted support for the functional repair of cartilage tissue. Current research on the role of HBOT in cartilage repair mainly focuses on *in vitro* models and animal models. To further clarify the potential mechanism of HBOT in cartilage regeneration, **Figure 3** demonstrates the core signaling pathways by which HBOT regulates the anoxia-inflammation-metabolic network in cartilage tissue.^22^ To further illustrate the scope of HBOT applications in knee-related disorders, **Table 1** provides an overview of current research on HBOT, including clinical and preclinical studies.^58^,^59^,^60^

**Table 1:** Summary of current studies on HBOT in knee-related disorders

Study	Design type	HBOT parameter	Main therapeutic effects
Juskovic et al.^58^	Animal study	100% O_2_, 2.4 ATA for 90 min daily over 2 wks	HBOT combined with allogenic adipose stem cells efficiently attenuated osteoarthritis progression, is an effective treatment for in intra-articular injection of monoiodoacetate-induced rat osteoarthritis
Ozturan and Akyuerek^59^	Retrospective clinical study	100% O_2_, 2.0–2.5 ATA for 60–90 min per session, typically over 10–20 sessions	Faster resolution of bone marrow edema with no severe adverse effects
Zhao et al.^60^	Randomized controlled clinical study	100% O_2_, 2.0 ATA for 60 min daily, beginning postoperatively and continuing for 10 sessions	Reduced postoperative edema and muscle injury markers, accelerated recovery, shortened hospital stay

ATA: Atmospheres absolute; HBOT: hyperbaric oxygen therapy; O_2_: oxygen.

Treatment pressure and duration matter in how well HBOT works for KOA.^61^,^62^ Yuan et al.^63^ looked at HBOT combined with low-intensity pulsed ultrasound. HBOT was administered at 100% O_2_, 2.0 ATA for 60 minutes daily. Both treatments improved the activity of chondrocytes in KOA. The combination increased the level of tissue inhibitor of metalloproteinase-1 and reduced the level of matrix metalloproteinase-3. Tissue inhibitor of metalloproteinase-1 is crucial for preventing enzymes, such as matrix metalloproteinase 3, from cartilage degradation. Keeping this balance helps protect the structure of the cartilage and the surrounding matrix. This protection is essential for maintaining joint health.

Although some studies have shown that the combined therapy can effectively improve the condition, the efficacy of HBOT alone for OA is not fully understood. Yılmaz et al.^64^ pointed out that HBOT has limited effects on reducing cartilage damage and preventing the development of KOA. These findings suggest that HBOT may be combined with other treatment methods to achieve better clinical efficacy.

## Future Prospects

### Currentd challenges

Despite the large number of studies on HBOT, clinical research remains limited, with most studies based on in vitro models and animal models. Current research has confirmed some mechanisms of HBOT, such as the regulation of vascular endothelial growth factor, inhibition of inflammatory factor release, promotion of cartilage matrix protein production, and reduction of cartilage-degrading enzyme activity. However, more specific molecular mechanisms still need to be investigated. This lack of well-defined mechanisms is one of the reasons for the absence of concrete theoretical support for clinical treatment protocols.

The scarcity of clinical trials means that there is insufficient data on the use of HBOT for KOA in real-world clinical settings. Key aspects, such as oxygen pressure, treatment duration, treatment frequency and overall treatment duration, remain unclear and vary widely between studies. The lack of standardized research makes it difficult to compare therapeutic results, while the absence of unified treatment guidelines hinders the widespread application of HBOT in clinical settings.

The long-term efficacy of hyperbaric oxygen therapy remains a subject of ongoing debate, indicating that improvements are still needed in this field. At the same time, we should not overlook the potential risks associated with HBOT, including but not limited to oxygen toxicity, barotrauma, and DNA damage following HBOT.^65^ The increase in oxidative stress levels could also pose potential harm to cartilage tissue.^66^ Therefore, how to safely and effectively use HBOT has become one of the most critical issues that we currently face in the clinical application of HBOT.

### Prospection

**Figure 4:**
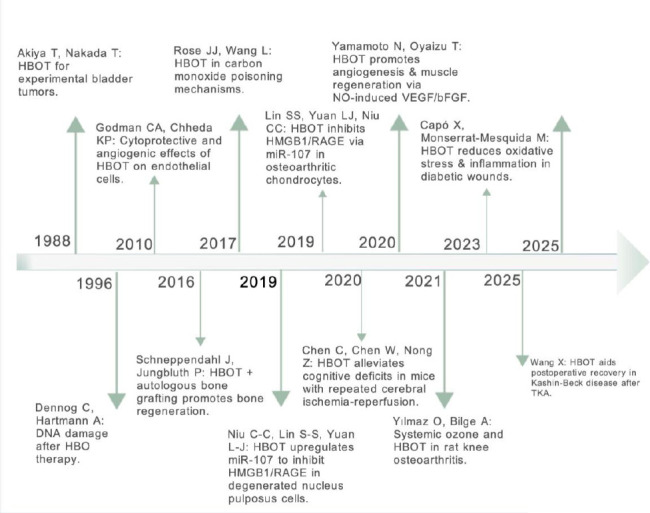
Chronology and key milestones of HBOT-related studies. Created with BioGDP.com.^22^ bFGF: Basic fibroblast growth factor; HBO: hyperbaric oxygen; HBOT: hyperbaric oxygen therapy; HMGB1: high mobility group protein B1; NO: nitric oxide; RAGE: receptor for advanced glycation end-products; TKA: total knee arthroplasty; VEGF: vascular endothelial growth factor.

As shown in **Figure 4**, the timeline of HBOT-related research clearly outlines important milestones from early experimental exploration to recent clinical application, vividly demonstrating the rapid understanding of its mechanism of action and treatment potential.^22^,^36^,^38^,^43^,^44^,^48^,^54^,^55^,^60^,^64^,^65^,^66^,^67^ Although HBOT has made its mark in mechanism research and initial clinical trials of KOA, the uneven efficacy among individuals is still the biggest stumbling block hindering its widespread clinical application. The speed of patient disease progression, the strength of treatment response, and the wide variety of biological characteristics urgently require us to shift from standardized treatment to tailor-made and individual plans. With the rapid integration of artificial intelligence (AI) into biomedical research, we have reason to expect that AI strategies will bring revolutionary and personalized breakthroughs to KOA’s HBOT protocols.

First, multi-modal data integration presents a critical foundation for constructing individualized HBOT response prediction models. By aggregating structured clinical data (e.g., age, body mass index, Kellgren-Lawrence grade), imaging biomarkers (e.g., cartilage thickness, subchondral bone microarchitecture on magnetic resonance imaging), molecular signatures (e.g., IL-1β, TNF-α, and HIF-1α), and patient-reported outcomes (e.g., Western Ontario and McMaster Universities Osteoarthritis Index, Visual Analogue Scale), machine learning models such as XGBoost or transformer-based deep learning architectures can be trained to: (1) distinguish HBOT responders from non-responders, (2) estimate optimal treatment duration and frequency, and (3) recommend adjunctive therapies such as non-steroidal anti-inflammatory drugs or platelet-rich plasma when appropriate.

Second, the incorporation of real-time therapeutic monitoring using mobile health devices (e.g., gait sensors, pain-tracking applications) and smart HBOT chambers can facilitate adaptive treatment modifications. AI algorithms may dynamically assess treatment response trends and recommend therapeutic adjustments, such as early termination, extension of HBOT courses, or escalation to combination therapy, based on observed efficacy plateaus or mechanistic mismatches (e.g., HIF-inactive OA subtypes).

Third, precise analysis based on genomics, metabolomics or microRNA expression may identify KOA subgroups that are sensitive to HBOT. AI-assisted molecular phenotyping analysis can promote pathophysiology-based KOA typing research, making interventions more precise and consistent with biological mechanisms.

Fourth, digital twin technology can create a virtual patient model to simulate the efficacy of HBOT under different parameters (oxygen pressure, duration, and frequency), thereby providing prophetic insights into cartilage protection and therapeutic response maps. This method may reduce repeated trial and error in clinical trials, optimize treatment plan design, and bring the overall safety and effectiveness of HBOT therapy to a higher level.

In summary, AI-enabled precision medicine offers a promising path to optimizing HBOT for KOA. From treatment indication screening to response prediction, real-time adjustment, and mechanistic matching, AI can provide full-process support for individualized HBOT strategies. Future efforts should focus on building high-quality multicenter datasets, validating predictive models in prospective clinical trials, and enhancing algorithmic transparency and clinical interpretability to move HBOT from empirical use toward a data-driven, evidence-based therapeutic modality.

There is still huge room for exploration in HBOT. Combining HBOT with other treatment methods may lead to a more comprehensive and effective treatment plan to address the multi-factorial nature of KOA. This not only promotes cartilage repair, but also reduces inflammation, controls pain and improves joint function, thereby improving treatment results. Continued research is crucial to explore the potential synergistic effects of HBOT when used in conjunction with other innovative therapies.

Although this review uses a strict data selection approach, its limitations must be acknowledged. At present, most research on HBOT still focuses on animals and *in vitro* models, and clinical trials are still in their early stages. There is a lack of high-quality randomized controlled trials to verify its long-term efficacy and safety. Standardized guidelines for treatment options have not yet been established, and comparability between different studies is limited, which hinders systematic evaluation of the effectiveness of HBOT. Although the need for personalized treatment is urgent, there is no clear diagnostic tool or standardized treatment process in clinical practice.

To sum up, this review reveals that HBOT has great potential in the treatment of KOA. By analyzing the pathogenesis of KOA, the principle of action and its impact of HBOT, this paper comprehensively expounds the mechanism of action and efficacy of HBOT based on animal and *in vitro* experimental models, demonstrating its broad prospects. The development of big data has laid a solid foundation for personalized medical services, and these advances have opened new possibilities for future medical decision-making.

## Data Availability

*Not applicable.*
